# Antibacterial Activity of *Solanum torvum* Leaf Extract and Its Synergistic Effect with Oxacillin against Methicillin-Resistant Staphyloccoci Isolated from Dogs

**DOI:** 10.3390/antibiotics11030302

**Published:** 2022-02-24

**Authors:** Duangdaow Khunbutsri, Nattakarn Naimon, Khomson Satchasataporn, Natnaree Inthong, Sarawan Kaewmongkol, Samak Sutjarit, Chanokchon Setthawongsin, Nattakan Meekhanon

**Affiliations:** Department of Veterinary Technology, Faculty of Veterinary Technology, Kasetsart University, Bangkok 10900, Thailand; cvtddk@ku.ac.th (D.K.); cvtnkn@ku.ac.th (N.N.); cvtkss@ku.ac.th (K.S.); cvtnri@ku.ac.th (N.I.); cvtswt@ku.ac.th (S.K.); cvtsms@ku.ac.th (S.S.); chanokchon_s@hotmail.com (C.S.)

**Keywords:** antimicrobials, dog, staphylococci, resistance

## Abstract

Methicillin-resistant staphylococci (MRS) have been considered a veterinary and public health threat that needs to be addressed, as they are known to cause serious infections, with limited therapeutic options. Thus, in this study, we aimed to examine the potential antibacterial activity of the leaf extract of *Solanum torvum* against MRS isolated from clinically healthy dogs. In total, seven *mecA*-positive *Staphylococcus* isolates tested in this study were identified using 16S rRNA gene sequencing, and all of them were classified as multidrug-resistant using disk diffusion tests. According to gas chromatography-mass spectrometry analysis, the main phytochemical components found in the leaf extract were hexadecanoic acid and its ethyl ester and 9,12,15-octadecatrienoic acid, ethyl ester, (Z,Z,Z). The minimum inhibitory concentration (MIC) breakpoints for the leaf extract against all tested isolates ranged from 2 to 16 mg/mL, while the MIC breakpoints for oxacillin were from 2 to 512 mg/L. Although varying effects were found, the positive effects of the leaf extract were most evident in combination with oxacillin. These results suggested that *S. torvum* leaf extract may complement classical antibiotics and may potentially drive the development of an effective therapeutic option for MRS.

## 1. Introduction

Although most staphylococci are commensal bacteria, some of them can cause serious infections of the skin and other tissues in both animals and humans. According to their ability to produce coagulase, staphylococci can be classified into the following two groups: coagulase-positive and coagulase-negative staphylococci. Coagulase-positive staphylococci, particularly *Staphylococcus aureus* and *Staphylococcus pseudintermedius* are commonly involved in infection in humans and companion animals, respectively, while coagulase-negative staphylococci (CoNS) are frequently related to nosocomial infection [1,2]. In the last few decades, the incidence of methicillin-resistant staphylococci (MRS) has seen a steady increase, which has led to it becoming a major concern worldwide. Since MRS can occur in companion animals and the transmission of MRS between animals and humans may potentially occur [3,4], MRS is considered to be a significant threat to public health. Methicillin resistance in staphylococci has mainly been associated with the *mecA* gene, which encodes a modified penicillin-binding protein 2a (PBP2a), causing a low binding affinity to β-lactam antibiotics [5]. In veterinary medicine, infections and colonization with MRS strains have been recognized as a serious problem because of their multidrug resistance and the possibility of transmission to humans. MRS strains are known to be resistant to several classes of antimicrobial agents, including β-lactam antibiotics, so therapeutic options are limited [6,7,8]. Therefore, traditional plant medicine may provide a potential alternative approach to treating and controlling the spread of MRS.

*Solanum torvum* (Swartz or turkey berry) is a small spiny tree widely distributed in India; China; Southeast Asian countries, including Thailand, Malaysia, and the Philippines; and tropical America. Different parts of this plant, particularly the fruits and leaves, produce antimicrobial effects and could therefore be used as medicinal plants [9,10,11]. Leaf extracts of *S. torvum* have been shown to effectively inhibit various pathogenic bacterial strains [12], although there is a lack of information regarding the antibacterial activity of *S. torvum* against antimicrobial-resistant bacteria. Due to the limited options for the treatment and control of MRS infection, the leaf extract of *S. torvum* is a potentially valuable candidate for the management of this public health threat.

In this study, we aimed to evaluate the antibacterial activity of the leaf extract of *S. torvum* against MRS isolated from clinically healthy dogs. The synergistic effects between the extract and oxacillin were also investigated.

## 2. Materials and Methods

### 2.1. Plant Preparation and Extraction

Leaves of *S. torvum* collected from the Nan province in the northern part of Thailand were used in this study. They were identified and authenticated at the Office of the Forest Herbarium, Department of National Parks, Wildlife and Plant Conservation, Thailand (code: BKF 186114). After air-drying at room temperature, the sampled leaves were ground to a fine powder. Then, approximately 600 g of the leaf powder was extracted with 5400 mL of 95% ethanol, as described in a previous study [12].

The leaf extract was dissolved in dimethyl sulfoxide (DMSO) (Merck, Germany) to a final concentration of 50% (*v*/*v*) and filtered through No. 1 Whatman filter paper to obtain the crude extract used for the examination of antibacterial activity. The phytochemical components of the crude extract were analyzed using GC-MS with a 6980 GC system (Agilent Technologies; Santa Clara, CA, USA), as described previously [12].

### 2.2. Bacterial Strains and Identification

In total, seven *mecA*-positive *Staphylococcus* isolates collected from the head (*n* = 2), mouth (*n* = 3), and nasal cavity (*n* = 2) of six clinically healthy dogs were used in this study. These three sites of sample collection are considered to be areas of high-frequency contact between dogs and owners. Ethical approval for the animal research was obtained from Kasetsart University (ACKU 01557). The bacterial isolates were cultured on mannitol salt agar (Himedia, India) and presumptively identified as *Staphylococcus* spp. by Gram staining and catalase tests. After DNA extraction using an E.Z.N.A.^®^ Bacterial DNA kit (Omega Bio-Tek, Doraville, GA, USA) following the manufacturer’s instructions, all isolates were definitely identified using 16S rRNA gene sequencing, as previously described [13], and the results were further analyzed using EzBioCloud (https://www.ezbiocloud.net/) (accessed on 13 May 2021) [14]. In addition, *S. aureus* ATCC 25923 was used as a quality control strain for antimicrobial susceptibility testing.

The detection of *mecA* was performed using PCR, as described previously [15], with slight modifications. Briefly, the primers used for the amplification of *mecA* were *mec*A-1 (5′-AAAATCGATGGTAAAGGTTGGC-3′) and *mec*A-2 (5′-AGTTCTGCAGTACCGGATTTGC-3′), giving a product of 533 bp. The conditions for PCR amplification were as follows: 94 °C for 2 min, followed by 30 cycles of 94 °C for 15 s, 55 °C for 30 s, and 72 °C for 30 s, and a final extension at 72 °C for 10 min. The PCR products were analyzed using gel electrophoresis. After staining with SYBR^®^ green (Sigma Aldrich, MO, USA), the bands of the amplified products were visualized using a gel documentation system. Positive and negative controls were included in all reactions.

### 2.3. Disk Diffusion Tests

Five representative antimicrobial agents from different classes—ciprofloxacin (5 µg), clindamycin (2 µg), erythromycin (15 µg), trimethoprim/sulfamethoxazole (1.25/23.75 µg), and tetracycline (30 µg) (Oxoid, Basingstoke, UK)—were used for antimicrobial susceptibility tests by disk diffusion, as recommended by the Clinical and Laboratory Standards Institute (CLSI) [16]. In addition, all isolates were tested for methicillin resistance using cefoxitin (30 µg) or oxacillin (1 µg) disk diffusion, as appropriate to the species. The test for detecting inducible clindamycin resistance (D-test) was performed in all isolates according to CLSI [16].

### 2.4. Microdilution Broth Susceptibility Assay

The determination of the minimum inhibitory concentration (MIC) and minimum bactericidal concentration (MBC) of crude extracts of *S. torvum* leaves and oxacillin was performed using the microdilution broth method [16] with slight modifications. Twelve twofold serial dilutions of the extract (256–0.125 mg/mL) and oxacillin (0.512–0.005 mg/L) were prepared in a 96-well microplate. To each well, 100 μL of each dilution of the leaf extract or oxacillin and 100 μL of bacterial suspension in Mueller–Hinton broth (MHB) (Oxoid, Basingstoke, UK) with a concentration of 1 × 10^6^ CFU/mL were added to produce a final bacterial number of approximately 1.5 × 10^5^ CFU per well. Sterility control with only MHB medium and positive control with MHB medium and bacterial inoculum were included in the experiment. In addition, we added DMSO as a negative control at equal concentrations (12.5–0.05% *v*/*v*) to those used to dilute the leaf extract. The plates were incubated at 37 °C for 24 h and the bacterial viability was examined by adding *p*-iodonitrotetrazolium chloride (INT) (Sigma-Aldrich, St. Louis, MO, USA). After incubation at 37 °C for 30 min, the color alteration was observed in the wells containing viable bacteria. Then, the MIC values were determined. The MIC and the more concentrated test dilutions were inoculated into Mueller–Hinton agar (Oxoid, Basingstoke, UK); then, the MBC values were further determined after an overnight incubation at 37 °C. The assays were independently performed in triplicate. 

### 2.5. Synergistic Interaction Analysis

To determine the potential synergistic antibacterial activity of the leaf extract of *S. torvum* and oxacillin, the checkerboard method was used [17]. Briefly, the leaf extract was diluted twofold in MHB along the vertical rows of the 96-well microplate, while oxacillin was cross-diluted horizontally by twofold serial dilution. Bacterial suspension was added into each well to produce a final concentration of 1.5 × 10^5^ CFU/mL. The plates were incubated at 37 °C for 24 h. After adding INT and incubating at 37 °C for 30 min, the bacterial growth was assessed by observing the color of the solution. The tests were carried out in triplicate.

Synergistic effects were determined based on the fractional inhibitory concentration index (FICI). The FICI was calculated as follows:

FIC oxacillin = MIC oxacillin in combination/MIC oxacillin alone;(1)

FIC leaf extract = MIC leaf extract in combination/MIC leaf extract alone; (2)

FICI = FIC oxacillin + FIC leaf extract.(3)

The FICI value was evaluated as follows: synergy (FICI < 0.5), partial synergy (0.5 ≤ FICI ≤ 0.75), additive (0.76 ≤ FICI ≤ 1), indifference (1 < FICI ≤ 4), or antagonism (FICI > 4) [18]. The averages of MICs obtained from three independent experiments were used for these equations.

### 2.6. Statistical Analyses

All tests were independently performed in triplicate. The differences between the data were analyzed using one-way ANOVA with repeated measures in SPSS version 28.0.1.0 (142) (IBM, Armonk, NY, USA). The *p*-values were considered statistically significant at ≤0.05.

## 3. Results and Discussion

### 3.1. Resistance Profiles of Bacterial Strains

As shown in Table 1, seven *mec*A-positive *Staphylococcus* isolates were identified as *Staphylococcus schleiferi* subsp. *schleiferi* (*n* = 1), *Staphylococcus epidermidis* (*n* = 2), *Staphylococcus intermedius* (*n* = 3), and *Staphylococcus pseudintermedius* (*n* = 1), using 16S rDNA sequencing. These staphylococcal species are commensals on skin and mucous membranes and can cause infections in both humans and animals [19,20,21,22].

According to the results from disk diffusion tests, all isolates were found to be resistant to at least three classes of antimicrobial agents and were therefore considered to be multidrug-resistant (MDR) bacteria [23]. Their resistance profiles are presented in Table 1. Among the isolates used in this study, those isolated from the nasal cavities and mouths of dogs tended to be resistant to most antimicrobial agents tested. One isolate from the nasal cavities of dogs (DN41) and two isolates from the mouths of dogs (DN11 and DN40) which were identified as *S. intermedius*, as well as one *S. pseudintermedius* isolate (DN73) from a dog’s mouth, were resistant to all tested antimicrobials. By D-test, two phenotypes were observed among the isolates tested in this study. *S. schleiferi* subsp. *schleiferi* (DN2) was erythromycin-sensitive and clindamycin-sensitive, indicating a susceptible phenotype, while the other six isolates showed resistance to both erythromycin and clindamycin, indicating a constitutive macrolide-lincosamide-streptogramin B (MLS_B_) phenotype.

Although information on methicillin-resistant coagulase-negative staphylococci (MRCoNS) in pets is rare compared with that on methicillin-resistant *S. aureus* (MRSA), these results suggested that a considerable amount of antimicrobial resistance was present in CoNS and other commensal bacteria in healthy pets. Since a high prevalence of MRS has previously been reported in healthy dogs [24] and the transfer of methicillin-resistant *S. pseudintermedius* (MRSP) among pets, humans, and the environment has evidently occurred [3], people who have close contact with pets would be at increased risk of transmission of these MRS bacteria.

MRS (including MRCoNS) are potentially a neglected risk to public health. Therefore, the use of natural extracts or combinations of extracts and existing antimicrobial agents may provide effective alternative options for the treatment and prevention of MRS bacteria.

### 3.2. Phytochemical Components of Leaf Extract

An ethanol extraction of *S. torvum* leaves was analyzed using gas chromatography-mass spectrometry (GC-MS). The phytochemical components of the extract and their potential biological activities are shown in Table 2. In total, 11 chemical compounds, including fatty acid ethyl esters, fatty acids, diterpenes, indoles, and alkanes, made up 60.12% of the total extract, as determined by GC fraction (Supplementary Figure S1). The major components were hexadecanoic acid, ethyl ester (11.13%), followed by hexadecanoic acid (8.63%) and 9,12,15-octadecatrienoic acid, ethyl ester, (Z,Z,Z) (7.47%). These compounds have also been identified in extracts of the fruit of *S. torvum* [25] and other plants [26,27,28]. The common biological activities of the abundant compounds of *S. torvum* leaf extract are antibacterial and anti-inflammatory. Consistent with a previous study, hexadecanoic acid, ethyl ester, and 9,12,15-octadecatrienoic acid, ethyl ester, (Z,Z,Z), were identified as major components of other leaf extracts which had potent activity against both Gram-positive and Gram-negative bacteria, as well as MRSA [28]. Hexadecanoic acid and its methyl and ethyl esters have previously been reported to exhibit antituberculotic activity in actinobacteria [29]. It is, therefore, suggested that the major phytochemical compounds (hexadecanoic acid, ethyl ester, hexadecanoic acid, 9,12,15-octadecatrienoic acid, ethyl ester, (Z,Z,Z) found in our leaf extract play a crucial role in bacterial inhibition.

The GC-MS results showed that neophytadiene was found twice, in peaks No. 4 and 10 (Table 2, Supplementary Figure S1). However, due to their different retention times, these two peaks were possibly related compounds with slightly different chemical structures.

### 3.3. Minimum Inhibitory Concentration (MIC) and Synergistic Interaction Analysis

The results from MIC and checkerboard analysis are presented in Table 3. All isolates were phenotypically confirmed as MRS (MIC of oxacillin ≥ 0.5 mg/L) [16]. They had MIC and minimum bactericidal concentration (MBC) values ranging from 2 to 512 mg/L for oxacillin, with the MIC and MBC values of *S. torvum* leaf extract ranging from 2 to 16 mg/mL and 8 to 64 mg/mL, respectively. Three *S. intermedius* isolates—DN11, DN40, and DN41—which were resistant to all antimicrobial agents tested also showed high MIC values for oxacillin. However, the leaf extract exhibited effective antibacterial activity against these three isolates, with an MIC range of 2–4 mg/mL. Similarly, the leaf extract of *S. torvum* has been reported to be effective against several pathogenic bacteria [12] and mycotoxigenic fungi [39].

**Figure 1:**
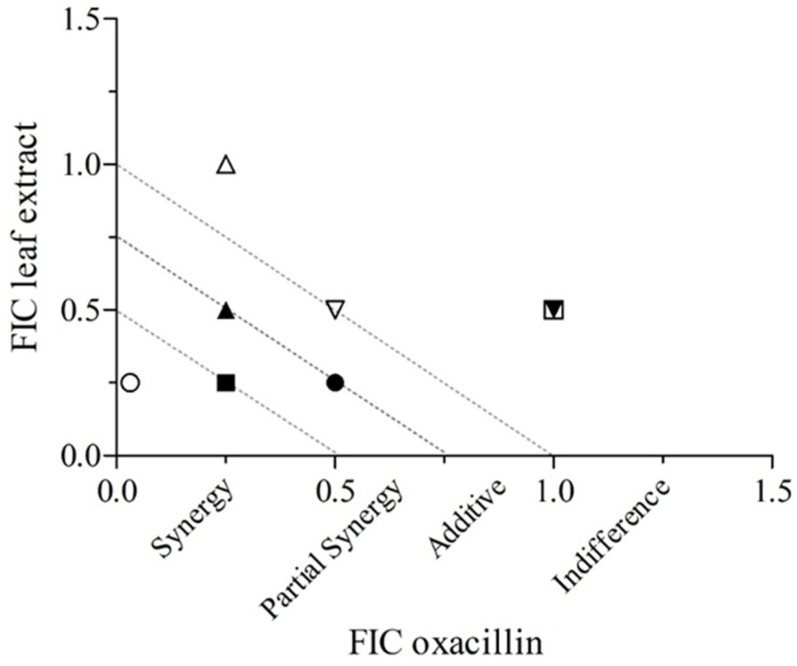
Isobolograms showing the synergy (FIC index ≤ 0.5), partial synergy (0.5 ≤ FICI ≤ 0.75), additive (0.76 ≤ FICI ≤ 1), and indifference (1 < FICI ≤ 4) effects of oxacillin and *S. torvum* leaf extract: interaction assays with oxacillin against methicillin-resistant staphylococci strains ▲ DN 2; ▼ DN 10; ● DN 11; ○ DN 18; ∆ DN 40; ∇ DN 41; ■ DN 73; □ *S. aureus* ATCC25923. Isobolograms were plotted using the GraphPad Prism, ver. 9.0, software to present the FIC index of the combinations.

The combination of oxacillin and *S. torvum* leaf extract exhibited varying antibacterial effects against the MRS strains tested (Table 3 and Figure 1). Although no antagonism was detected, only limited effects were found for 2/7 isolates. Synergistic and partially synergistic effects were observed for 4/7 isolates, while an additive effect was noted in one isolate of *S. intermedius*. The positive interaction was observed against all staphylococcal species used in this study—*S. schleiferi*, *S. epidermidis*, *S. intermedius*, and *S. pseudintermedius*—indicating the potential of the use of *S. torvum* leaf extract in combination with common antibiotics to counteract MRS.

The cytotoxic effects of crude extract of *S. torvum* leaves were previously evaluated in vitro. Since the antimycobacterial activity of *S. torvum* leaves has been observed, the cytotoxic potential of *S. torvum* leaf extract was investigated against a human fetal lung fibroblast cell line [40]. Although the notable cytotoxicity of several compounds of *S. torvum* aerial parts against human cancer cell lines was identified [41,42], the *S. torvum* leaf extract was found to be safe in a human fetal lung fibroblast cell line, supporting the traditional use of these leaves to treat respiratory tract disorders [40]. 

Due to the limited therapeutic options available for the treatment of MRS infections, novel alternatives, particularly phytopharmaceuticals, have been attracting increasing interest. The antimicrobial activity of several natural product extracts, and the synergistic effects between the extracts and antimicrobial agents, have been investigated. The antibacterial activity of various leaf extracts and their synergy with antibiotics against MRSA have previously been observed [43,44]. The combination of methicillin or penicillin G and essential oil has recently been reported to be effective against MRSA [45]. In this study, we demonstrated the potent activity of *S. torvum* leaf extract against MRCoNS and MRSP. The major components, hexadecanoic acid and its ethyl esters, are likely to be the bioactive compounds detected in our crude extract. However, further study regarding the antibacterial activity, pharmacological properties, and cytotoxic effects of these purified compounds is required to elucidate the antibacterial mechanism and the possibility of their use in vivo.

## 4. Conclusions

Methicillin-resistant staphylococci known to possess multidrug-resistant traits were isolated from healthy dogs. In this study, we investigated the antimicrobial activity of the crude extract of *Solanum torvum* leaves against the tested isolates and identified positive interactions between the extract and oxacillin. The results highlight *S. torvum* as a promising therapeutic option for methicillin-resistant staphylococci.

## Figures and Tables

**Table 1:** Information about the isolates and their resistance profiles.

Isolate	Bacterial Species Identified by 16s rDNA Sequencing	Similarity (%)/ Completeness (%)	Source	Resistant Profiles
DN 2	*Staphylococcus schleiferi* subsp. *schleiferi*	99.86/100	Head	CIP, TE, OX
DN 10 ^1^	*Staphylococcus epidermidis*	100/100	Head	DA, E, TE, OX
DN 11 ^1^	*Staphylococcus intermedius*	99.93/100	Mouth	CIP, DA, E, SXT, TE, OX
DN 18	*Staphylococcus epidermidis*	100/100	Nasal cavities	DA, E, TE, OX
DN 40	*Staphylococcus intermedius*	99.93/100	Mouth	CIP, DA, E, SXT, TE, OX
DN 41	*Staphylococcus intermedius*	99.93/100	Nasal cavities	CIP, DA, E, SXT, TE, OX
DN 73	*Staphylococcus pseudintermedius*	100/99.5	Mouth	CIP, DA, E, SXT, TE, OX
QC strain	*Staphylococcus aureus* ATCC25923	-	-	-

^1^ The isolates were recovered from the same dog. CIP, ciprofloxacin (5 µg); DA, clindamycin (2 µg); E, erythromycin (15 µg); SXT, trimethoprim/sulfamethoxazole (1.25/23.75 µg); TE, tetracycline (30 µg); OX, cefoxitin (30 µg) or oxacillin (1 µg).

**Table 2:** Chemical composition of ethanolic extract of *S. torvum* leaves.

Peak No.	Chemical Compound	Formula	Retention Time (min)	Area (%)	Biological Activity
1	Tetradecane	C_14_H_30_	26.28	0.90	Antimicrobial [30]
2	Octadecane	C_18_H_38_	38.25	1.58	Antimicrobial [30]
3	Heptadecane, 8-methyl-	C_18_H_38_	49.45	1.87	Anticancer, pest repellent, sex pheromone [31]
4	Neophytadiene	C_20_H_38_	51.30	4.58	Antioxidant, antibacterial, antifungal [32]
5	Hexadecanoic acid	C_16_H_32_O_2_	59.13	8.63	Antibacterial, anti-inflammatory, antioxidant [33,34]
6	Hexadecanoic acid, ethyl ester	C_18_H_36_O_2_	59.44	11.13	Antibacterial, anti-inflammatory [26]
7	Linoleic acid ethyl ester	C_20_H_36_O_2_	67.15	6.07	Antibacterial, antifungal [35]
8	9, 12, 15-Octadecatrienoic acid, ethyl ester, (Z,Z,Z)-	C_20_H_34_O_2_	67.40	7.47	Anti-inflammatory, antimicrobial, antioxidant [28]
9	Heptadecanoic acid, 15-methyl-, ethyl ester	C_20_H_40_O_2_	68.82	4.52	Antidiabetic, antioxidant [36]
10	Neophytadiene	C_20_H_38_	69.66	1.85	Antioxidant, antibacterial, antifungal [32]
11	5-Methyl-2-phenyl-1H-Indole	C_15_H_13_N	83.62	5.05	Antimicrobial, antifungal [37]
12	1,1-Dicyano-2-methyl-4-(p-cyanophenyl) propene	C_13_H_9_N_3_	84.65	6.47	Antifungal, insecticidal [38]

**Table 3:** Synergistic activity of the *S. torvum* leaf extract with oxacillin against methicillin-resistant staphylococci.

Isolate	Bacterial Strains	MBC		MIC Alone		MIC Combination		FIC Index	Interpretation
		Oxacillin (mg/L)	Extract (mg/mL)	Oxacillin (mg/L)	Extract (mg/mL)	Oxacillin (mg/L)	Extract (mg/mL)		
DN 2	*S. schleiferi* subsp. *schleiferi*	8	64	2	16	0.5	8	0.75	Partial synergy
DN 10 ^1^	*S. epidermidis*	64	32	64	4	64	2	1.5	Indifference
DN 11 ^1^	*S. intermedius*	256	8	256	4	128	1	0.75	Partial synergy
DN 18	*S. epidermidis*	128	32	64	8	2	2	0.28	Synergistic
DN 40	*S. intermedius*	512	16	512	2	128	2	1.25	Indifference
DN 41	*S. intermedius*	512	16	256	4	128	2	1	Additive
DN 73	*S. pseudintermedius*	2	32	2	4	0.5	1	0.5	Synergy
Control	*S. aureus* ATCC25923	1	16	0.5	16	0.5	8	1.5	Indifference

^1^ The isolates were recovered from the same dog. The values are presented as the average from three independent experiments.

## Data Availability

The authors confirm that the data supporting the findings of this study are available within the article.

## Supplementary Materials

The following are available online at https://www.mdpi.com/article/10.3390/antibiotics11030302/s1: Figure S1: Chemical composition of ethanolic extract of *S. torvum* leaves.
