# Supplementary figures and images for: Antibacterial Activity of Solanum torvum Leaf Extract and Its Synergistic Effect with Oxacillin against Methicillin-Resistant Staphyloccoci Isolated from Dogs
Source: Antibiotics (Basel). 2022 Feb 24;11(3):302. doi: 10.3390/antibiotics11030302 (PMC8944679; doi:10.3390/antibiotics11030302)

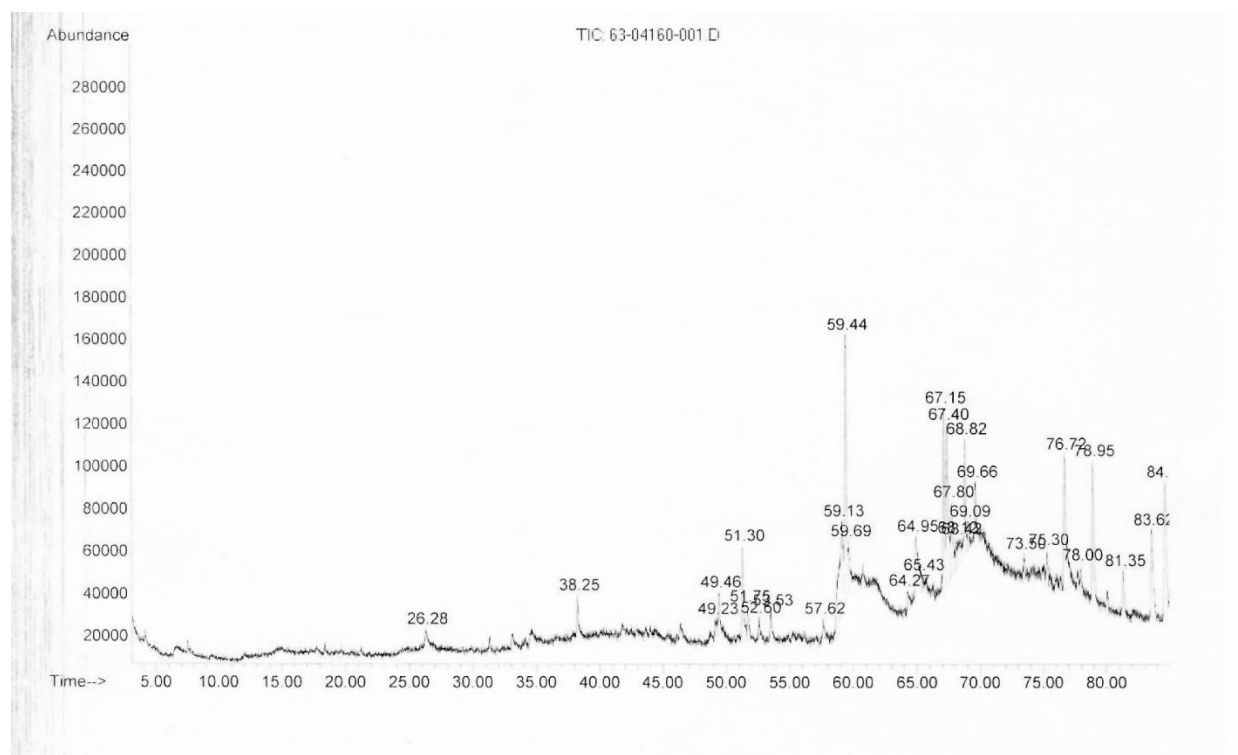

**Supplementary Figure S1.** GC-MS chromatogram of ethanolic extract of *Solanum torvum* leaves.

Supplement: Supplementary file 1 [file antibiotics-11-00302-s001.zip › antibiotics-1568654-supplementary.pdf]
